# Pericapsular nerve group block combined with lateral femoral cutaneous nerve block for hip surgery: a meta-analysis

**DOI:** 10.3389/fpain.2025.1723417

**Published:** 2026-01-12

**Authors:** Fan Yang, Qiang Tang, Bing Wang, Le Chen

**Affiliations:** 1 Department of Pain Management, Huanggang Central Hospital, Huanggang, China; 2 School of Nursing, Huanggang Polytechnic College, Huanggang, China

**Keywords:** analgesia, hip surgery, lateral femoral cutaneous nerve block, pericapsular nerve group block, systematic review and meta-analysis

## Abstract

**Objective:**

The combination of the pericapsular nerve group (PENG) block and lateral femoral cutaneous nerve (LFCN) block has garnered increasing attention as a postoperative analgesic strategy following hip surgery. Nevertheless, the clinical efficacy of this approach remains a subject of ongoing debate. Through a meta-analysis, the effects of the combined PENG and LFCN block in patients who underwent hip surgery were investigated.

**Methods:**

We conducted a systematic search of relevant clinical randomized controlled trials (RCTs) available in English via online databases and grey literature resources. Quantitative analyses were performed to assess pain scores, time to first rescue analgesia, consumption of rescue analgesics, incidence of quadriceps weakness, time to first ambulation, and postoperative nausea and vomiting (PONV) to comprehensively evaluate the effects of the combined PENG and LFCN block in patients after hip surgery.

**Results:**

A total of 12 RCTs involving 823 patients were included in this study. The findings from the quantitative synthesis indicate that the combined PENG and LFCN block significantly decreases postoperative pain scores at rest (*p* < 0.001) and during movement (*p* = 0.021), increases the time to first rescue analgesia (*p* < 0.001), and reduces the consumption of rescue analgesics (*p* < 0.001). Additionally, this combination decreases the incidence of quadriceps weakness (*p* < 0.001), shortens the time to first ambulation (*p* < 0.001), and decreases the incidence of PONV (*p* = 0.020).

**Conclusion:**

The combined PENG and LFCN block has favourable clinical efficacy for postoperative analgesia in hip surgery patients and is recommended for use. However, more high-quality, large-scale RCTs are needed to further validate our findings.

**Systematic Review Registration:**

https://www.crd.york.ac.uk/PROSPERO/view/CRD420251142338, PROSPERO CRD420251142338.

## Introduction

Hip surgery is a prevalent procedure in the field of orthopaedics and includes various interventions, such as joint repair and replacement. Given its demonstrable efficacy and significant impact on patient outcomes, the demand for such procedures has been steadily increasing (1). However, the majority of patients who undergo hip surgeries, including hip arthroplasty, experience moderate to severe postoperative pain (2). In accordance with the principles of enhanced recovery after surgery (ERAS) protocols, patients should be encouraged to mobilize early after anaesthesia through the use of multimodal analgesia (including regional techniques) to improve outcomes and reduce the length of hospital stay (3, 4). While opioids provide effective analgesia, their significant associated side effects pose potential clinical risks, particularly for the elderly population. In contrast, regional nerve blocks not only alleviate pain but also reduce opioid consumption, thereby minimizing related adverse effects (5). Consequently, regional nerve blocks offer remarkable analgesic benefits within the framework of ERAS protocols. The neural architecture of the hip joint is complex, with the anterior two-thirds predominantly innervated by nociceptive fibres from the femoral nerve and the obturator nerve and the posterior one-third supplied by proprioceptive fibres from the sciatic nerve (6). Notably, the majority of pain receptors are located in the anterior and medial compartments of the joint and are governed primarily by the lumbar plexus (7). This anatomical complexity prevents the achievement of complete unilateral lower limb anaesthesia through a single peripheral technique. Pain management strategies often require anaesthetic approaches for the lumbar plexus and its branches. However, these approaches can result in quadriceps muscle motor blockade, leading to limitations in mobility and extended rehabilitation times. In contrast to traditional nerve blocks, motor-sparing peripheral nerve blocks facilitate early ambulation, reduce postoperative pain scores, and decrease the demand for opioids (8, 9). Therefore, the advantages of motor-sparing peripheral nerve blocks align with the principles of ERAS and have emerged as an effective analgesic strategy for patients undergoing hip surgery.

The pericapsular nerve group (PENG) block is an innovative technique that involves the injection of a local anaesthetic within the plane between the iliopsoas tendon and the iliopubic eminence. This technique selectively targets the articular branches of the femoral and obturator nerves, with the PENG block exhibiting greater selectivity for the obturator nerve. Furthermore, the PENG block can reduce motor blockade by modulating the volume and concentration of the injected anaesthetic agent, thereby preserving motor function (10–12). The lateral femoral cutaneous nerve (LFCN) provides sensory innervation to the lateral aspect of the thigh. Pain in this region is a significant contributor to postoperative discomfort following hip surgeries, as it is closely associated with the surgical incision and represents a substantial component of postoperative pain (13). However, a considerable number of hip surgeries are now being performed using the anterior approach. This may limit the efficacy of the LFCN block. Consequently, the clinical value of a combined PENG and LFCN block remains uncertain.

In light of these findings, we conducted a meta-analysis to quantitatively evaluate and comprehensively analyse the efficacy and safety of the combined application of the pericapsular nerve group block and lateral femoral cutaneous nerve block following hip surgery. The aim of this investigation is to provide a valuable reference for clinical judgement.

## Methods

### Database search and literature acquisition

Our study adhered to the Preferred Reporting Items for Systematic Reviews and Meta-Analyses (PRISMA) guidelines (14), and our protocol was preregistered on the PROSPERO website under ID CRD420251142338. A thorough literature search was conducted across the PubMed, Cochrane Library, Directory of Open Access Journals, Web of Science, and Embase databases, supplemented by a search for grey literature in the OpenGrey and ProQuest Dissertations & Theses databases, to identify studies that explored the postoperative efficacy of the combined PENG and LFCN block in patients undergoing hip surgery. Specific MeSH search terms were employed, either individually or in combination (as exemplified by the search strategy in Supplementary Table S1 for PubMed), followed by a preliminary comparison of candidate literature and duplicate records across the different databases for initial screening. The initial inclusion criteria included the availability of the full text in English and a publication date prior to October 2025. Subsequent screening was performed on the basis of the title, abstract, and full text of the identified studies.

### Inclusion and exclusion criteria

The inclusion criteria were as follows: (1) randomized controlled trials (RCTs), including original articles, letters, or short communications; (2) an experimental group that received the combined PENG and LFCN block under ultrasound guidance as the sole intervention under comparable baseline analgesia conditions; (3) clinical studies that focused specifically on hip surgery; and (4) studies that delineated a defined postoperative observation period and provided relevant data. The exclusion criteria included the following: (1) observational studies, cross-sectional studies, and case series or case reports; (2) lack of clarity regarding the interventions in the experimental group; (3) basic medical research conducted on animal models; (4) the absence of full text in English or inability to obtain the full text; (5) failure to provide any relevant parameter data; and (6) the presence of duplicate publications of the same sample.

### Data extraction

Two researchers independently extracted relevant data from the included studies, and any discrepancies were resolved through group discussion. General information about the studies, such as publication date and sample size, and important information concerning methodology were systematically recorded in a predesigned table. The primary objective of this study was to elucidate the postoperative analgesic effects of the combined PENG and LFCN block. To achieve this goal, we extracted data on relevant pain metrics, including visual analogue scale (VAS) and numerical rating scale (NRS) scores measured at rest and during movement, time to first rescue analgesia, and the consumption of rescue analgesics for quantitative analysis. The secondary objective was to assess the impact of the PENG block in conjunction with the LFCN block on postoperative motor recovery and safety. Accordingly, we analysed data concerning the incidence of quadriceps weakness and the time to first ambulation to quantitatively evaluate the recovery of postoperative functional mobility. Additionally, the incidence of postoperative nausea and vomiting (PONV) served as a safety evaluation metric for the combined PENG and LFCN block. Apart from the data on the time to first rescue analgesia, time to first ambulation and consumption of rescue analgesics, we extracted other data at various follow-up time points and subsequently aggregated them and stratified them by equivalent follow-up durations. Continuous variables are summarized as means with standard deviations. When only medians with ranges or interquartile ranges (IQRs) were provided, the data were subjected to conversion using established formulas (15, 16).

### Assessment of the methodological quality and evidence for outcome quality

The study design and methodology of the included studies were evaluated for quality using the Cochrane Risk of Bias Assessment Tool (17). This assessment specifically addressed the following domains: selection bias, performance bias, detection bias, attrition bias, and reporting bias. For the final quantitative evidence, the Grading of Recommendations Assessment, Development and Evaluation (GRADE) system (18) was employed to assess the level of evidence. The evaluations of quality and evidence recommendations were independently conducted by the two aforementioned researchers, with discrepancies managed in a manner consistent with the previously outlined approach.

### Statistical analysis

Given the potential for variability in assessment methods and units for continuous variables, the results of synthesized data analyses are presented as standardized mean differences (SMDs) accompanied by corresponding 95% confidence intervals (CIs). Binary outcomes are summarized using the relative risk (RR) and corresponding 95% CI. The extent of heterogeneity was calculated to inform the choice of analytical model; a random effects model was applied in instances of substantial heterogeneity (*I*² ≥ 50%), while a fixed effects model was utilized when heterogeneity was low (*I*² < 50%). Meta-regression analyses and Egger's test were conducted to quantitatively assess outcome variability and detect publication bias using the STATA software package (version 15.0). The evaluation of the level of evidence was performed using GRADE Profiler (version 3.6).

## Results

### Characteristics of the included studies

**Figure 1:**
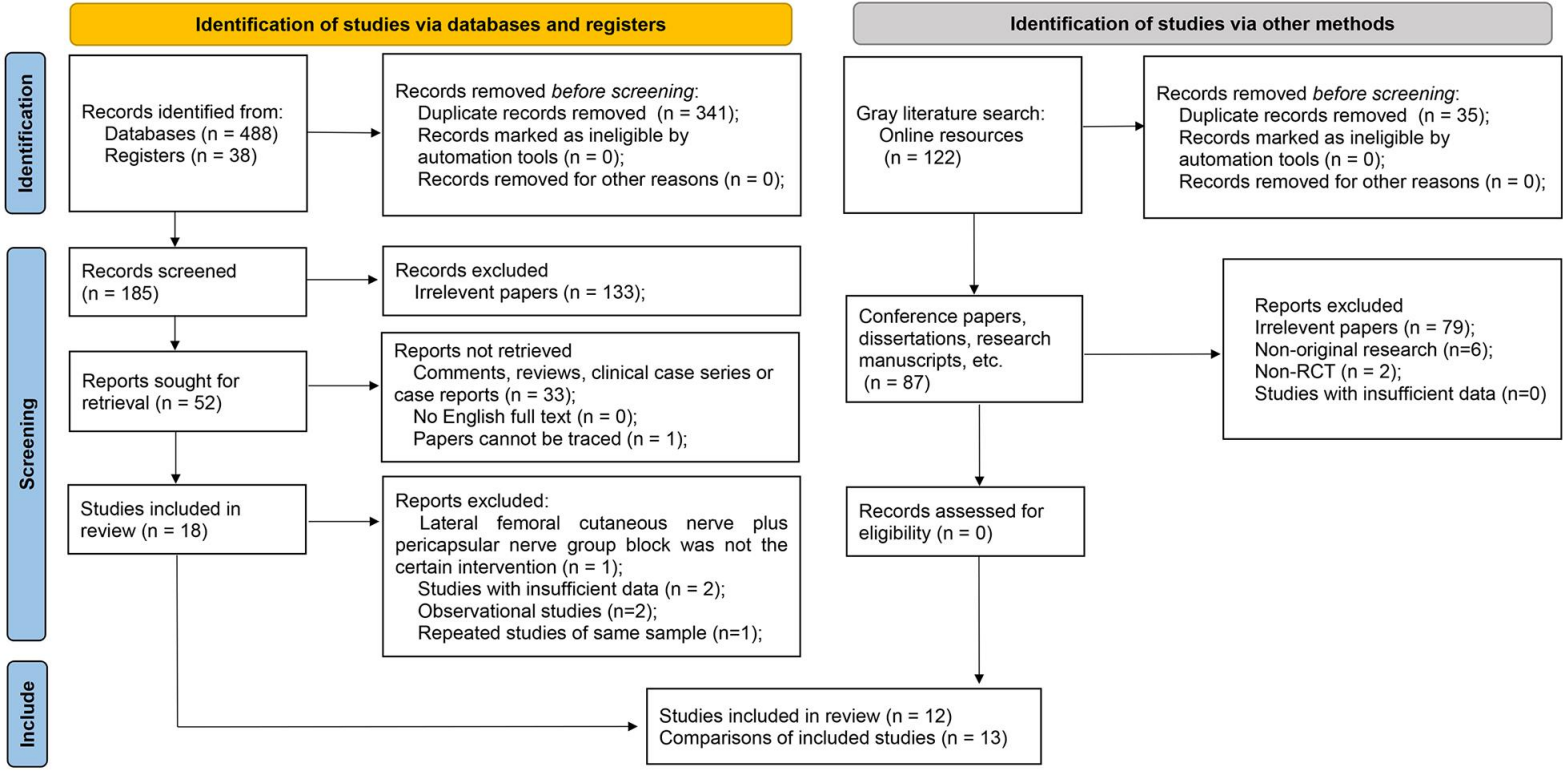
Flow diagram illustrating the study selection process for the meta-analysis.

Following an initial database search, a meticulous review of titles, abstracts, and full texts culminated in the inclusion of 12 RCTs containing 13 comparisons involving a total of 823 patients for the final quantitative analysis (19–30) (Figure 1). All the studies included in the analysis were published within the past five years. One of the studies was a three-arm RCT (26), in which the group receiving the combined PENG and LFCN block served as the experimental group and two comparisons were performed, while the remaining studies were two-arm trials (Table 1).

**Table 1:** Characteristics of included studies.

Author	Year	Country/region	Design	Arm	Sample szie	Timing of block	Volume and percentage of anesthetics	Surgical procedure	Elective/emergency	Hip arthroplasty approach	Method of anesthesia	Follow-up	Available Parameters
Aslan et al. (19)	2025	Turkey	RCT	2	80	Preoperative	PENG (0.25% bupivacaine, 25 mL) + LFCN (0.25% bupivacaine, 5 mL) vs. QL (0.25% bupivacaine, 30 mL)	Total hip arthroplasty	Unclear	Unclear	Spinal anesthesia	24 h	Quantity of rescue analgesia (morphine) Number of quadriceps weakness Incidence of PONV
Buffoli et al. (20)	2025	Italy	RCT	2	62	Preoperative	PENG (0.5% ropivacaine, 20 mL) + LFCN (0.5% ropivacaine, 10 mL) vs. ESP (0.5% ropivacaine, 30 mL)	Total hip arthroplasty	Elective	Posterolateral approach	Spinal anesthesia	48 h	Quantity of rescue analgesia (morphine) VAS/NRS (at rest/movement) Number of quadriceps weakness Incidence of PONV
Dang et al. (21)	2024	Vietnam	RCT	2	60	Unclear	PENG (0.25% ropivacaine, 20 mL) + LFCN (0.25% ropivacaine, 10 mL) vs. PCA (1 mg/mL, 10 mg/4 h)	Hip replacement	Unclear	Unclear	Unclear	72 h	Incidence of PONV
Hay et al. (22)	2024	USA	RCT	2	101	Preoperative	PENG (0.25% ropivacaine, 20 mL) + LFCN (0.25% ropivacaine, 10 mL) vs. QL (0.25% ropivacaine, 30 mL)	Total hip arthroplasty	Unclear	Unclear	Spinal/General anesthesia	72 h	VAS/NRS (at rest/movement)
Jadon et al. (23)	2022	India	RCT	2	60	Preoperative	PENG (0.5% ropivacaine, 25 mL) + LFCN (0.5% ropivacaine, 5 mL) vs. PENG (0.5% ropivacaine, 30 mL)	Fractured hip surgery	Unclear	Unclear	Spinal anesthesia	24 h	Time to first rescue analgesia Quantity of rescue analgesia (tramadol)
Liang et al. (24)	2023	China	RCT	2	92	Preoperative	PENG (0.33% ropivacaine, 20 mL) + LFCN (0.33% ropivacaine, 10 mL) vs. FIC (0.33% ropivacaine, 30 mL)	Total hip arthroplasty	Elective	Posterolateral approach	General anesthesia	48 h	Time to first ambulation Incidence of PONV
Liu et al. (25)	2024	China	RCT	2	78	Preoperative	PENG (0.375% ropivacaine, 35 mL) + LFCN (0.375% ropivacaine, 5 mL) vs. FIC (0.375% ropivacaine, 40 mL)	Arthroscopic hip surgery	Unclear	Unclear	General anesthesia	48 h	Number of quadriceps weakness Time to first ambulation Quantity of rescue analgesia (ketorolac tromethamine) Incidence of PONV
Şeker et al. (26)	2025	Turkey	RCT	3	60	Preoperative	PENG (0.25% bupivacaine, 20 mL) + LFCN (0.25% bupivacaine, 5 mL) vs. PENG (0.25% bupivacaine, 20 mL) vs. No treatment	Fractured hip surgery	Elective	Unclear	Spinal anesthesia	24 h	Quantity of rescue analgesia (morphine) VAS/NRS (at rest/movement) Time to first rescue analgesia Time to first ambulation
Svraka et al. (27)	2024	Bosnia and Herzegovina	RCT	2	66	Preoperative	PENG (0.25% bupivacaine, 20 mL) + LFCN (0.25% bupivacaine, 10 mL) vs. No treatment	Total hip arthroplasty	Elective	Posterolateral approach	Spinal anesthesia	36 h	Incidence of PONV
Tian et al. (28)	2025	China	RCT	2	60	Unclear	PENG (0.4% ropivacaine, 20 mL) + LFCN (0.4% ropivacaine, 3 mL) vs. FIC (0.4% ropivacaine, 40 mL)	Total hip arthroplasty	Unclear	Unclear	General anesthesia	48 h	VAS/NRS (at rest/movement) Number of quadriceps weakness Quantity of rescue analgesia (sufentanil) Incidence of PONV
Vetrone et al. (29)	2025	Italy	RCT	2	58	Preoperative	PENG (0.5% ropivacaine, 20 mL) + LFCN (0.5% ropivacaine, 10 mL) vs. FIC (0.5% ropivacaine, 20 mL)	Total hip arthroplasty	Elective	Direct anterior approach	Spinal anesthesia	48 h	VAS/NRS (at rest/movement) Incidence of PONV
Yoo et al. (30)	2024	Korea	RCT	2	46	Preoperative	PENG (0.375% ropivacaine, 20 mL) + LFCN (0.375% ropivacaine, 5 mL) vs. No treatment	Total hip arthroplasty	Elective	Unclear	General anesthesia	48 h	Quantity of rescue analgesia (fentanyl)

FIC, fascia iliaca compartment; PENG, pericapsular nerve group; LFCN, lateral femoral cutaneous nerve; QL, quadratus lumborum; ESP, erector spinae plane; PCA, patient-controlled analgesia; VAS, visual analog scale; NRS, numerical rating scale; PONV, postoperative nausea and vomiting.

With respect to the procedural methodology for the combined PENG and LFCN block, all the studies explicitly indicated that the procedures were performed under ultrasound guidance, with the PENG block administered first, followed immediately by the LFCN block. The anaesthetic region for the PENG block involved the neural plane surrounding the iliopsoas tendon. The injection site for the LFCN block was located in the region of the anterior superior iliac spine, sartorius and tensor fasciae latae, including the subcutaneous tissue in between. All injections utilized the in-plane technique. With respect to the timing of nerve block application, ten RCTs clearly established that the combined PENG and LFCN block was administered prior to surgery, with only two studies failing to provide relevant information. Concerning the selection of anaesthetic agents for the PENG and LFCN block, in three RCTs 0.25% bupivacaine was utilized, with volumes of 20 to 25 mL for the PENG block and 5 to 10 mL for the LFCN block. In the remaining nine studies, ropivacaine was employed at concentrations varying from 0.25% to 0.5%, with total volumes ranging from 20 to 35 mL for the PENG block and 3 to 10 mL for the LFCN block. With respect to the nerve block techniques utilized in the control groups, in one study, the PENG block was applied alone; in four studies, the fascia iliaca compartment (FIC) block was implemented; in two studies, the quadratus lumborum (QL) block was employed; and in one RCT, the erector spinae plane (ESP) block was employed for control purposes. In the remaining four RCTs, the control groups did not receive any nerve blockade interventions; furthermore, in three of these four studies, analgesic measures beyond the routine analgesic treatment administered to all participants were not employed (Table 1).

In terms of surgical procedures, nine of the included RCTs involved patients undergoing total hip arthroplasty. Among these, in three RCTs, the posterolateral approach was clearly employed; in one RCT, the direct anterior approach was utilized, and in the remaining studies, relevant information was not provided. Six RCTs reported on elective procedures, while the remaining studies did not specify this information. With respect to the anaesthesia techniques used for hip surgery, in six RCTs, spinal anaesthesia was used; in four RCTs, general anaesthesia was employed; and in one RCT, both spinal and general anaesthesia were used, whereas in the remaining RCT, the anaesthesia method was not specified. The duration of postoperative observation varied from 24 to 72 h (Table 1).

### Quality assessment

**Figure 2:**
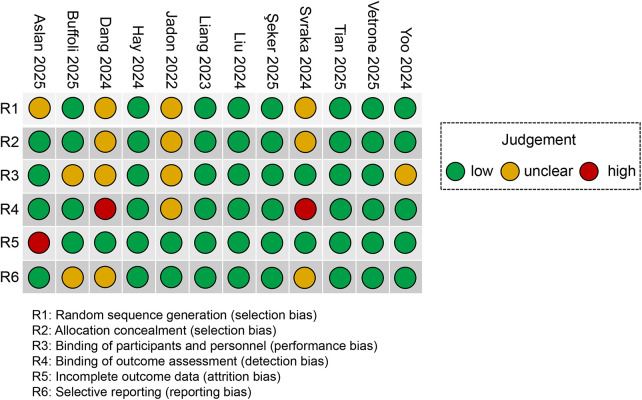
Assessment of bias pertaining to each risk of bias item in the included studies.

Among the included studies, in eight, random allocation was implemented, and in nine RCTs, random concealment was used. In seven studies, a double-blind experimental design was employed; however, two studies presented a risk of nonblinding. In terms of the double-blind approach, the anaesthesiologists administered nerve blocks independently from the researchers to ensure complete separation and noninvolvement in subsequent observations and data analyses, thereby preserving investigator blinding. Additionally, the anaesthesiologists followed the established protocol to conduct ultrasound scans of the targeted nerve sites for all patients and executed sterile skin preparation along with subcutaneous anaesthesia at the designated insertion points to ensure patient blinding. Furthermore, in certain studies, the anaesthesiologists performed nerve blocks immediately after anaesthesia was induced and just prior to the commencement of the surgical procedure, which not only minimized patient discomfort but also contributed to maintaining the blinding effect for the patients. With respect to attrition and reporting bias risks, eleven RCTs demonstrated clear data integrity, and nine studies were found to be free from selective reporting bias. Overall, the quality of the study designs among the included studies can be regarded as acceptable, although potential biases still warrant further analysis and discussion (Figure 2).

### Analysis of postoperative pain in patients

**Figure 3:**
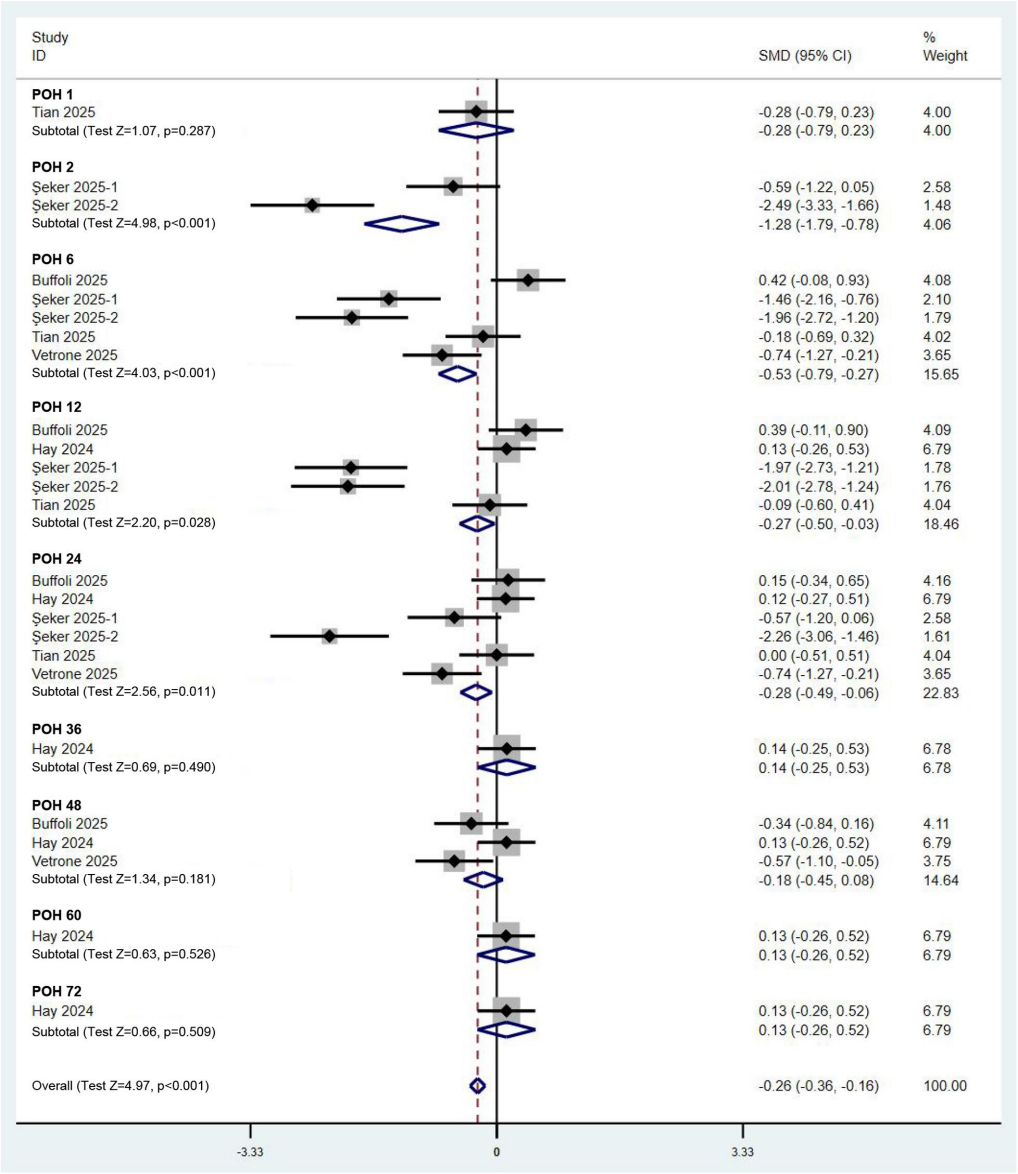
Forest plot comparing pain scores at rest between the combined PENG and LFCN block group and the control group. POH, postoperative hour.

**Figure 4:**
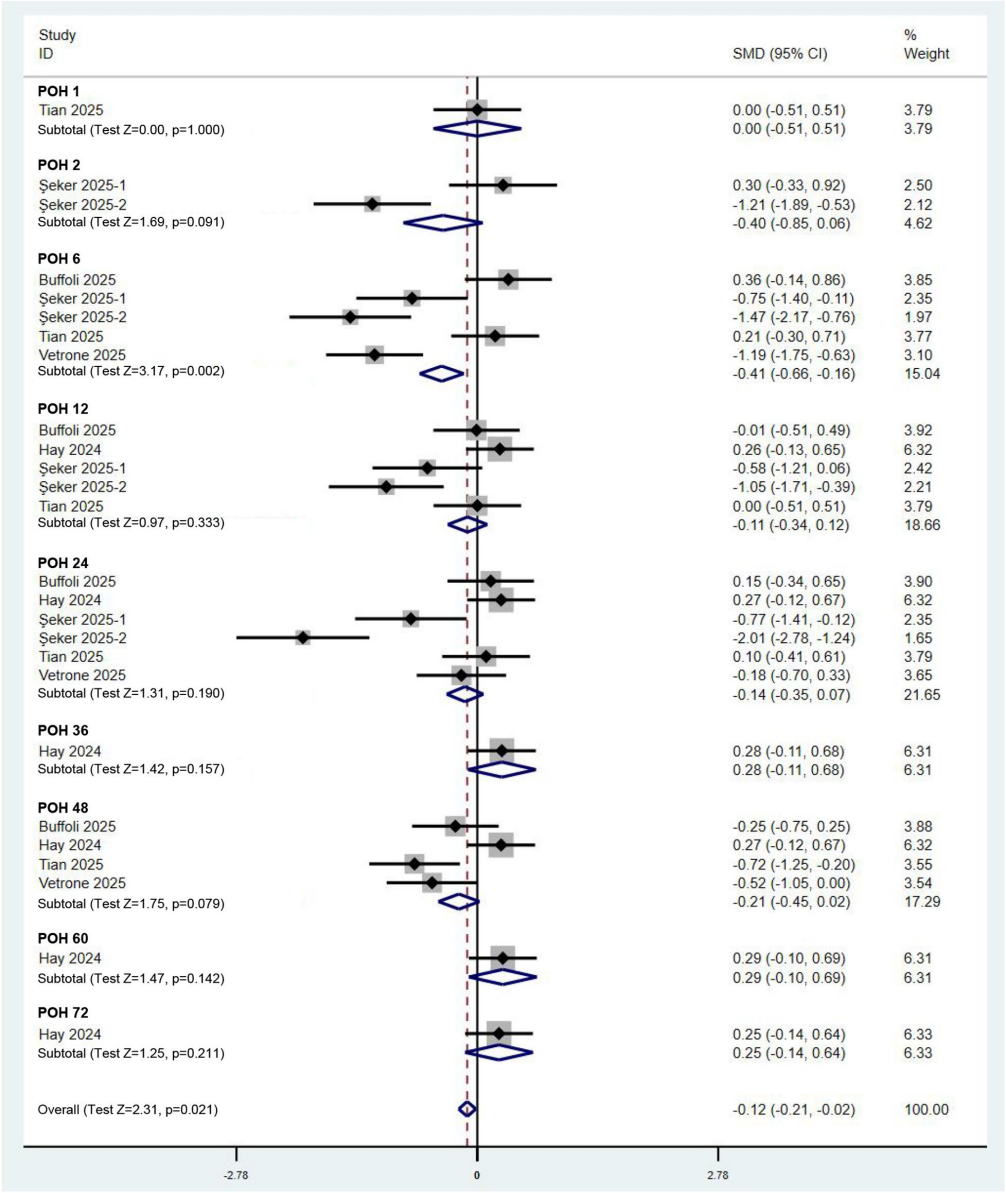
Forest plot comparing pain scores during movement between the combined PENG and LFCN block group and the control group.

To elucidate the impact of the combined PENG and LFCN block on postoperative pain in hip surgery patients, we first conducted a quantitative analysis of postoperative pain scores. A total of five RCTs, encompassing six comparisons, provided data on pain scores. According to a random effects model (*I*^2^ = 86.0%), compared with other analgesic methods, the combined PENG and LFCN block significantly reduced postoperative pain scores at rest [SMD (95% CI) = −0.26 (−0.36, −0.16), *p* < 0.001]. Notably, the primary time window for effective pain relief occurred between 2 and 24 h postoperatively (Figure 3). In terms of dynamic pain scores, the combined PENG and LFCN block also resulted in a significant reduction in postoperative pain [SMD (95% CI) = −0.12 (−0.21, −0.02), *p* = 0.021], as analysed using a random effects model (*I*^2^ = 78.9%) (Figure 4).

**Figure 5:**
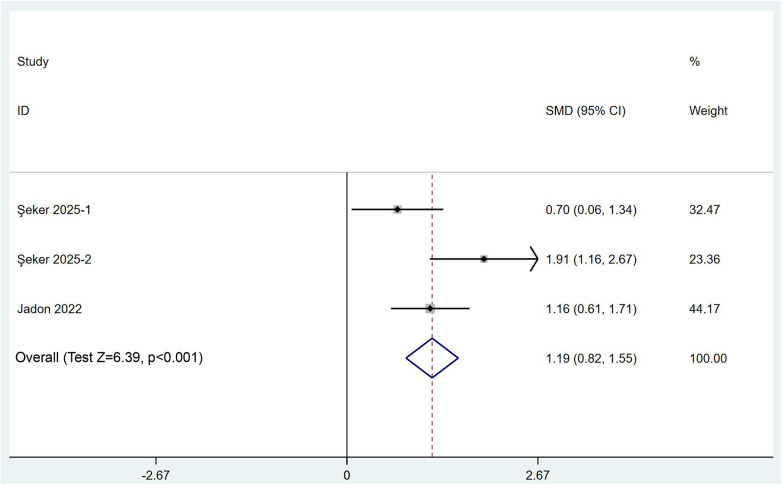
Forest plot comparing the time to first rescue analgesia between the combined PENG and LFCN block group and the control group.

**Figure 6:**
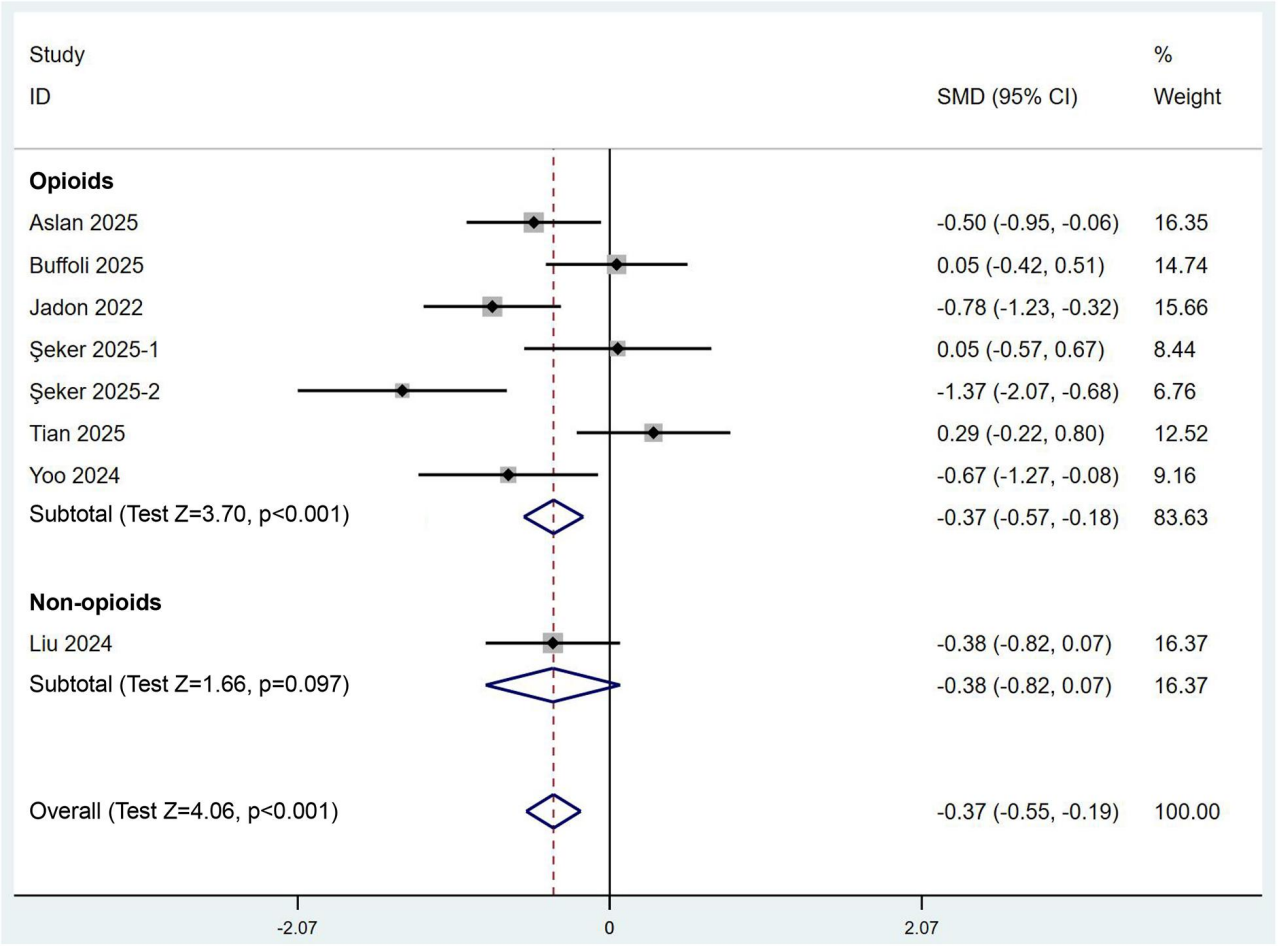
Forest plot illustrating the consumption of rescue analgesics in the combined PENG and LFCN block group and the control group.

We subsequently quantitatively aggregated the data on postoperative rescue analgesia usage. We commenced our analysis by comparing the time to first use of rescue analgesics. Two RCTs encompassing three comparisons provided pertinent data, and the results derived from a random-effects model (*I*^2^ = 65.2%) suggested that the combined PENG and LFCN block significantly prolonged the duration of initial rescue analgesic administration [SMD (95% CI) = 1.19 (0.82, 1.55), *p* < 0.001] (Figure 5). With respect to the utilization of rescue analgesics, seven RCTs involving a total of 446 participants contributed relevant data; in six of the trials, opioids were employed, while in the remaining trial, nonsteroidal anti-inflammatory drugs (NSAIDs) were utilized. Quantitative analysis, which was based on a stratified analysis and was performed using a random-effects model (*I*² = 70.5%), revealed that the combined PENG and LFCN block significantly reduced the need for rescue analgesia [SMD (95% CI) = −0.37 (−0.55, −0.19), *p* < 0.001], particularly opioids [SMD (95% CI) = −0.37 (−0.57, −0.18), *p* < 0.001] (Figure 6).

### The impact of the combined PENG and LFCN block on postoperative mobility recovery

**Figure 7:**
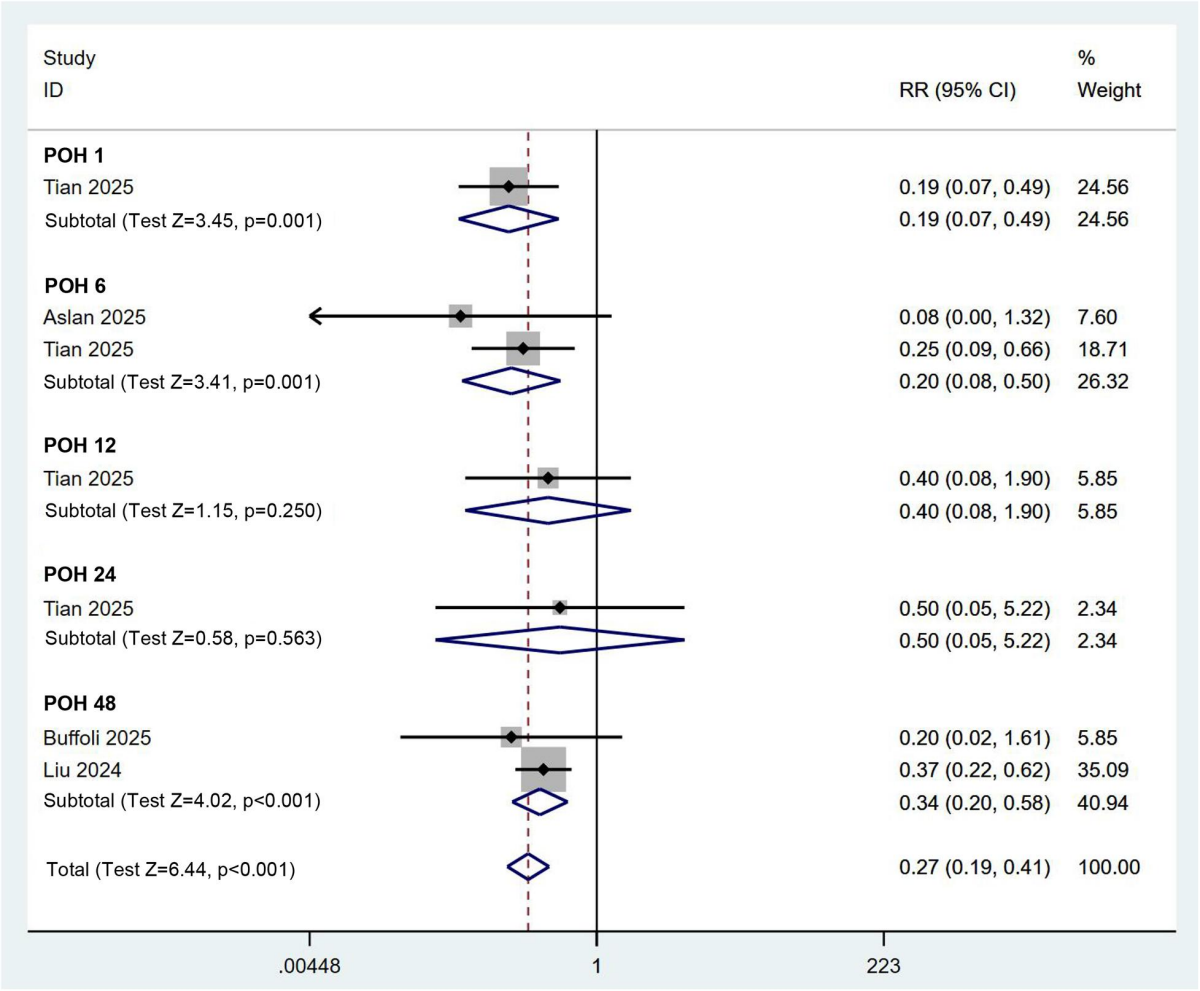
Forest plot depicting the incidence of quadriceps weakness between the combined PENG and LFCN block group and the control group.

**Figure 8:**
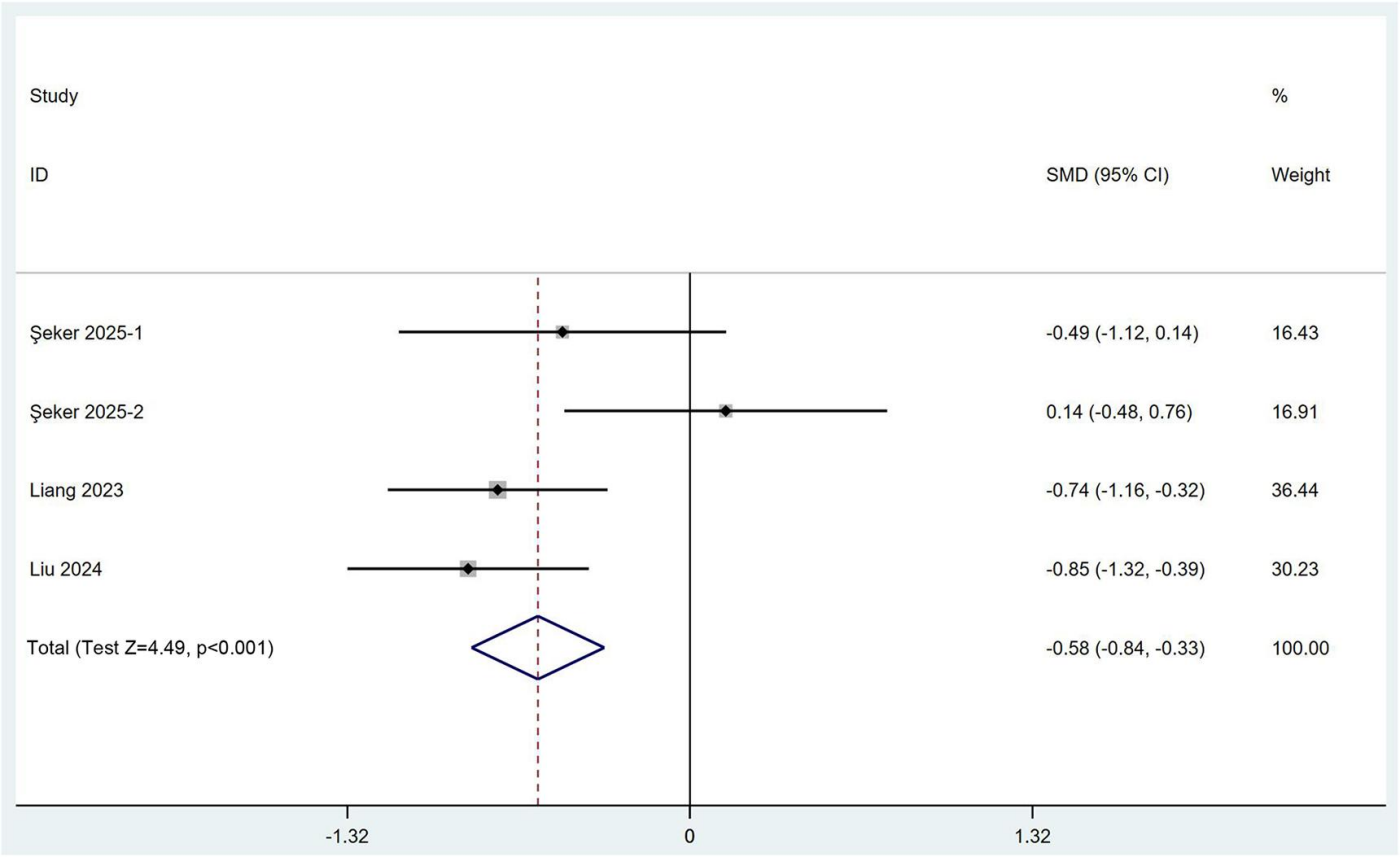
Forest plot demonstrating the time to first ambulation for the combined PENG and LFCN block group and the control group.

To determine whether the combined PENG and LFCN block influences postoperative mobility recovery, we assessed the incidence of quadriceps weakness and the time to first ambulation. Four RCTs involving 280 participants reported data on postoperative quadriceps weakness. Using a fixed effects model (*I*^2^ = 0.0%), the meta-regression analysis indicated that the combined PENG and LFCN block significantly reduced the incidence of quadriceps weakness [RR (95% CI) = 0.27 (0.19, 0.41), *p* < 0.001] (Figure 7). In addition, a quantitative analysis based on three RCTs comprising four comparisons and 230 patients revealed that, according to a random effects model (*I*^2^ = 57.7%), the combined PENG and LFCN block significantly shortened the time to first ambulation for patients [SMD (95% CI) = −0.58 (−0.84, −0.33), *p* < 0.001] (Figure 8). These findings suggest that the combined approach may facilitate enhanced mobility recovery for patients.

### Comparative analysis of PONV

**Figure 9:**
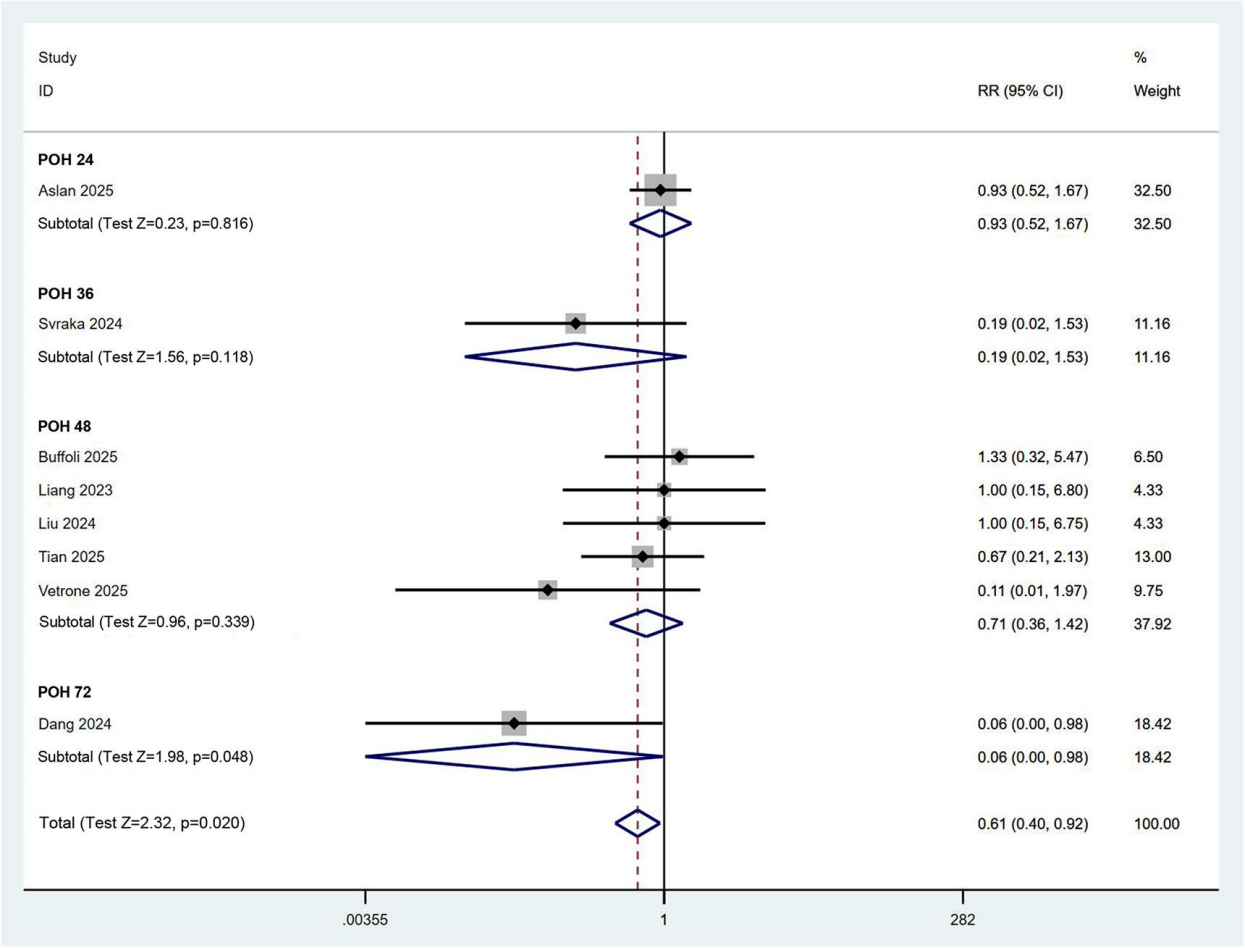
Forest plot showing the incidence of postoperative nausea and vomiting in the combined PENG and LFCN block group and the control group.

Owing to the heterogeneous nature of postoperative adverse events and the lack of standardized criteria, we employed the incidence of PONV as a primary indicator to assess the safety of the combined PENG and LFCN block. A total of eight RCTs involving 556 patients reported the incidence of PONV. According to a fixed effects model (*I*^2^ = 22.7%), the quantitative comparison of binary variables indicated that the application of the combined PENG and LFCN block significantly reduced the incidence of PONV [RR (95% CI) = 0.61 (0.40, 0.92), *p* = 0.020]. Although this effect may not have been prominently evident in the early postoperative period, it became significantly apparent after 48 h postsurgery (Figure 9). These results suggest that the combined PENG and LFCN block is highly safe and may contribute to enhanced postoperative comfort for patients.

### Reccomendation of evidence for outcomes and publiacation bias

Given the potential risk of bias inherent in the quality assessments mentioned above, we employed Egger's test to quantitatively evaluate publication bias across various parameters. Our analyses revealed significant data bias in the evaluation of postoperative pain scores (Supplementary Figures S1A,B), whereas no substantial publication bias was detected in the remaining parameters (Supplementary Figures S1C–G). Finally, on the basis of the quality of evidence assessment, the recommendation levels for pain scores and the incidence of PONV were determined to be moderate, while all the other indicators were classified as high (Table 2). Consequently, the overall findings have considerable reference value.

**Table 2:** The quality and recommendation of the evidence according to the GRADE system.

Outcomes	Anticipated comparative risks (95% CI)^a^		Relative effect (95% CI)	Significant (yes/no)	No. of participants (studies)	Quality of the evidence (GRADE)^b^
	Assumed risk (PENG block + LFCN block)	Corresponding risk (others)				
VAS/NRS (at rest)	The mean VAS/NRS is 1.37	The mean VAS is 0.63 higher (0.48 higher to 0.78 higher)	–	Yes	341 (5 RCTs)	⊕⊕⊕ Moderate^c^
VAS/NRS (at movement)	The mean VAS/NRS is 2.78	The mean VAS is 0.32 higher (0.30 higher to 0.35 higher)	–	Yes	341 (5 RCTs)	⊕⊕⊕ Moderate^c^
Time to first rescue analgesia	The mean time is 167.31	The mean time is 476.3 longer (459.10 longer to 493.50 longer)	–	Yes	120 (2 RCTs)	⊕⊕⊕⊕ High
Quantity of rescue analgesia	The mean quantity is 50.00	The mean quantity is 71.77 higher (71.39 higher to 72.15 higher)	–	Yes	446 (7 RCTs)	⊕⊕⊕⊕ High
Number of quadriceps weakness	70 per 1,000	280 per 1,000 (230 to 330)	0.27 (0.19, 0.41)	Yes	280 (4 RCTs)	⊕⊕⊕⊕ High
Time to first ambulation	The mean time is 17.38	The mean time is 3.48 longer (0.50 longer to 6.47 higher)	–	Yes	230 (3 RCTs)	⊕⊕⊕⊕ High
Incidence of PONV	100 per 1,000	150 per 1,000 (80 to 220)	0.61 (0.40, 0.92)	Yes	556 (8 RCTs)	⊕⊕⊕ Moderate^d^

High quality (⊕⊕⊕⊕): Further research is very unlikely to change our confidence in the estimate of effect.

Moderate quality (⊕⊕⊕): Further research is likely to have an important impact on our confidence in the estimate of effect and may change the estimate.

Low quality (⊕⊕): Further research is very likely to have an important impact on our confidence in the estimate of effect and is likely to change the estimate.

Very low quality (⊕): We are very uncertain about the estimate.

a The basis for the assumed risk is the mean control group risk across studies. The corresponding risk (and its 95% confidence interval) is based on the assumed risk in the comparison group and the relative effect of the intervention (and its 95% CI).

b GRADE Working Group grades of evidence.

c Publication bias detected (−1).

d Risk of bias from unblinding (−1).

### Subgroup analysis

On the basis of the aforementioned results, we found considerable heterogeneity in the quantitative analysis of certain indicators, and Egger's test results suggested a potential risk of bias in the pain score outcomes. To further investigate the effects of the combined PENG and LFCN block across various dimensions, we conducted relevant subgroup analyses. In terms of the nerve block techniques employed in the control groups, compared with no treatment, the combined PENG and LFCN block significantly reduced pain scores, thereby contributing substantially to the primary outcomes, particularly in the context of dynamic pain scores. Nonetheless, despite these findings, the combination demonstrated its own advantages relative to other forms of nerve blocks or analgesic methods. In subgroup analyses of patients receiving bupivacaine and ropivacaine, it appeared that the population receiving bupivacaine exhibited a more pronounced analgesic benefit from the combination, whereas those treated with ropivacaine appeared to experience enhanced postoperative functional recovery and safety. In the subgroup analysis based on the type of anaesthesia administered, we categorized the studies into those involving patients who received general anaesthesia and those involving patients who received spinal anaesthesia. The analgesic effect of the combination therapy was not significantly evident in patients who received general anaesthesia; however, it may exhibit certain benefits in relation to postoperative mobility recovery. Conversely, this trend appeared to be reversed in patients who received spinal anaesthesia. Furthermore, in the subgroup analysis by surgical technique, both minimally invasive arthroscopic procedures and other surgical interventions resulted in favourable analgesic effects and postoperative recovery outcomes when the combined PENG and LFCN block was administered. These findings suggest that the analgesic effectiveness of this combined approach is applicable across various surgical modalities. Importantly, however, despite the subgroup analyses conducted according to different items, heterogeneity in each subgroup, particularly with regard to pain scores and the need for rescue analgesia, remained unmitigated (Table 3).

**Table 3:** Results of subgroup analyses.

Subgroup		Intervention (control group)		Anesthetics for PENG block and LFCN block		Method of anesthesia		Surgical procedure	
Item		No treatment	The other interventions	Bupivacaine	Ropivacaine	General anesthesia	Spinal anesthesia	Arthroscopic surgery	Other surgeries
Pain score at rest	No. of comparisons	1	5	2	4	1	4	–	6
	*I* ^2^	0.0%	73.1%	76.4%	48.0%	0.0%	88.3%	–	86.0%
	SMD (95% CI)	−2.16 (−2.56, −1.77)	−0.12 (−0.23, −0.02)	−1.53 (−1.78, −1.27)	−0.02 (−0.13, 0.09)	−0.14 (−0.39, 0.12)	−0.67 (−0.82, −0.51)	–	−0.26 (−0.36, −0.16)
	*P*	<0.001	0.023	<0.001	0.707	0.286	<0.001	–	<0.001
Pain score at movement	No. of comparisons	1	5	2	4	1	4	–	6
	*I* ^2^	21.9%	64.4%	74.1%	61.0%	48.3%	78.9%	–	78.90%
	SMD (95% CI)	−1.40 (−1.75, −1.05)	−0.01 (−0.11, 0.10)	−0.87 (−1.11, −0.64)	0.04 (−0.06, 0.15)	−0.08 (−0.30, 0.15)	−0.47 (−0.62, 0.32)	–	−0.12 (−0.21, −0.02)
	*P*	<0.001	0.914	<0.001	0.418	0.516	<0.001	–	0.021
Quantity of rescue analgesia	No. of comparisons	2	6	3	5	3	5	1	7
	*I* ^2^	56.0%	65.5%	78.1%	71.3%	68.6%	74.5%	100.0%	76.20%
	SMD (95% CI)	−0.97 (−1.42, −0.52)	−0.19 (−0.37, −0.01)	−0.54 (−0.86, −0.22)	−0.20 (−0.40, 0.00)	−0.10 (−0.35, 0.15)	−0.46 (−0.69, −0.23)	−0.38 (−0.82, 0.07)	−0.29 (−0.47, −0.10)
	*P*	<0.001	0.044	0.001	0.045	0.438	<0.001	0.097	0.002
Number of quadriceps weakness	No. of comparisons	0	4	1	3	2	2	1	3
	*I* ^2^	–	0.0%	0.0%	0.0%	0.0%	0.0%	0.0%	0.00%
	RR (95% CI)	–	0.27 (0.19, 0.41)	0.08 (0.00, 1.32)	0.29 (0.20, 0.43)	0.30 (0.20, 0.44)	0.13 (0.02, 0.69)	0.37 (0.22, 0.62)	0.23 (0.13, 0.39)
	*P*	–	<0.001	0.077	<0.001	<0.001	0.017	<0.001	<0.001
Time to first ambulation	No. of comparisons	1	3	2	2	2	2	1	3
	*I* ^2^	100.0%	0.0%	48.4%	0.0%	0.0%	48.4%	100.0%	61.90%
	SMD (95% CI)	0.14 (−0.48, 0.76)	−0.73 (−1.01, −0.45)	−0.17 (−0.61, 0.27)	−0.79 (−1.10, −0.48)	−0.79 (−1.10, −0.48)	−0.17 (−0.61, 0.27)	−0.85 (−1.32, −0.39)	−0.47 (−0.77, −0.16)
	*P*	0.664	<0.001	0.446	<0.001	<0.001	0.446	<0.001	0.003
Incidence of PONV	No. of comparisons	1	7	2	6	3	4	1	7
	*I* ^2^	0.0%	14.5%	55.5%	21.6%	0.0%	37.5%	0.0%	33.80%
	RR (95% CI)	0.19 (0.02, 1.53)	0.66 (0.43, 1.02)	0.57 (0.13, 2.56)	0.50 (0.26, 0.95)	0.80 (0.33, 1.92)	0.70 (0.43, 1.16)	1.00 (0.15, 6.25)	0.59 (0.38, 0.91)
	*P*	0.118	0.059	0.462	0.033	0.618	0.170	1.000	0.017

## Discussion

On the basis of the results of the quantitative analysis derived from the meta-regression comparisons, our findings indicated that the combined PENG and LFCN block significantly reduced postoperative pain scores, prolonged the time to first administration of rescue analgesics, decreased the consumption of rescue analgesics, shortened the time to first postoperative ambulation, and decreased the incidence of quadriceps weakness and PONV in patients who underwent hip surgery. Therefore, in light of this evidence-based conclusion, we assert that the concomitant application of the combined PENG and LFCN block can yield substantial clinical benefits.

One of the primary advantages of the PENG block is its suitability for patients who experience acute or chronic pain due to hip fractures while they are in a supine position; furthermore, it has a protective effect on motor function by selectively targeting the articular branches of the femoral and obturator nerves (11). Additionally, the PENG block is not only utilized as an alternative regional anaesthesia technique for managing acute pain in hip fracture patients but has also been expanded to provide postoperative analgesia following elective hip surgeries (31, 32). Some studies even suggest that the PENG block may be effective for procedures such as venous ligation and dissection (33). Conversely, the lateral femoral cutaneous nerve has a distribution that facilitates pain management in the context of lateral surgical incisions. Consequently, the LFCN block is particularly well suited for analgesia in anterior–lateral thigh surgeries, such as the open reduction and internal fixation of hip fractures, total hip arthroplasty, and hemiarthroplasty (34). However, reports on the standalone application of the LFCN block are currently limited, as it is primarily employed as an adjunctive block in the context of hip surgery, often in combination with techniques such as the PENG block or FIC block.

Our findings confirmed the primary objective of this study: the combined PENG and LFCN block significantly reduced postoperative pain scores and the consumption of rescue analgesics and prolonged the time to first administration of rescue analgesics in patients undergoing hip surgery. Notably, the effective duration for pain suppression achieved by the combined PENG and LFCN block was primarily concentrated within the first 24 h after surgery. Considering the pharmacokinetics and the timing of pain onset, these results suggest that the maximal effects of the combined PENG and LFCN block occurs within the first 24 h following surgery. Previous reports have demonstrated that the application of the PENG block significantly decreases pain scores at 24 h after total hip arthroplasty while also notably reducing opioid consumption, with analgesic efficacy comparable to that of local infiltration analgesia (35, 36). These findings align with the temporal context of our results, suggesting that the isolated application of the PENG block can exert significant effects. However, a recent network meta-analysis indicated that among various standalone neuroblocking techniques, the PENG block demonstrates suboptimal analgesic efficacy compared with other methods in patients undergoing total hip arthroplasty (37). These observations further underscore that while the PENG block is effective when used alone, it may not represent the optimal analgesic strategy. This highlights the critical role of incorporating the LFCN block into postoperative analgesia for hip surgery, thereby emphasizing the necessity of using the PENG block in conjunction with the LFCN block to enhance pain management. Nonetheless, while the PENG block can effectively address capsular pain and the LFCN block can alleviate discomfort at the incision site, neither technique is capable of mitigating pain associated with the femoral component where the prosthesis is implanted. This limitation further indicates that the efficacy of the combined PENG and LFCN block is confined to specific areas.

In terms of postoperative functional recovery, our results indicate that the combined PENG and LFCN block significantly reduces the incidence of postoperative quadriceps weakness and notably shortens the time to the first mobilization after surgery. These findings suggest that the integration of the PENG block and LFCN block can markedly facilitate the rehabilitation of patients following hip surgery. While some studies have shown that, during the PENG block, the anaesthetic may inadvertently infiltrate the femoral nerve directly through the psoas major muscle or that the proximity of the puncture needle to the pubic muscle could facilitate the spread of the anaesthetic to the main trunk of the femoral nerve through the gap between the pubic muscle and the psoas major, thereby causing motor block (38), such occurrences can be technically mitigated. For instance, under ultrasound guidance, the needle can be accurately rotated to ensure that the tip penetrates the fascial layer of the psoas major. This approach allows the anaesthetic to diffuse effectively within the plane between the tendon of the psoas major and the iliopubic eminence. From a nerve-targeting perspective, the PENG block, as a regional anaesthesia technique, focuses primarily on the articular branches of the femoral nerve and obturator nerve. Consequently, the limited scope of anaesthesia helps preserve postoperative lower limb motor function. Although the anterior hip capsule is a primary source of pain in the hip joint, considering that incision-related pain can restrict early postoperative mobilization is crucial. Therefore, the adjunctive use of the LFCN block is likely to provide a more comprehensive analgesic effect, thereby accelerating the postoperative recovery of mobility.

We selected PONV as a key indicator of anaesthesia-related safety. Our results indicate that the combined PENG and LFCN block is associated with fewer complications, suggesting that, while effective, it also has a favourable safety profile. The anaesthetic scope of both the PENG block and LFCN block is relatively limited, as they are specifically targeted to local sensory nerves, resulting in minimal systemic effects, including a restricted influence on the gastrointestinal system. Furthermore, on the basis of our results, we demonstrated that the combined PENG and LFCN block significantly reduced the need for rescue analgesics, particularly with respect to opioids (Figure 6). Opioids are known to potentially induce nausea and vomiting, and this effect may be even more pronounced in patients who are experiencing their first exposure to such medications (39). Additionally, the earlier resumption of mobility and ambulation may facilitate the restoration of gastrointestinal function, which could further aid in alleviating opioid-induced PONV.

The results of the subgroup analysis indicate that compared with no treatment, the combined PENG and LFCN block has significant analgesic effects, which is in line with our expectations. While this combination also shows considerable advantages over other analgesic methods, no significant difference in pain scores during movement was observed. This finding could be attributed to the inclusion of patients in the control group who received other neuroblocking approaches. Although the combined PENG and LFCN block is effective, its performance may be less pronounced than that of other neuroblocking techniques. For instance, the control group included patients who received the ESP block and QL block, and recent findings from an RCT suggest no significant difference exists in analgesic efficacy between the ESP block and PENG block (40). Additionally, the analgesic effects of the PENG block alone do not appear to surpass those of the QL block (41). Furthermore, compared with the FIC block, the PENG block has superior analgesic effects; however, it falls short regarding improvements in the dynamic pain score (42). While this study incorporates a combination approach with the LFCN block, the aforementioned factors may influence the overall effectiveness of this combined approach to varying degrees. On another note, in the subgroup analysis focusing on anaesthetic selection and the method of surgical anaesthesia, the combined PENG and LFCN block appeared to yield better analgesic outcomes in patients receiving bupivacaine and in those opting for spinal anaesthesia. Conversely, the advantages related to recovery of movement do not seem as pronounced in this subgroup. For patients undergoing hip surgery, compared with ropivacaine, bupivacaine has been shown to provide superior analgesic effects (43, 44). Similarly, compared with general anaesthesia, spinal anaesthesia tends to offer greater benefits in terms of both analgesic efficacy and recovery (45). Therefore, we hypothesize that the use of bupivacaine and spinal anaesthesia collectively reduces the baseline pain levels of patients postoperatively, allowing the combined PENG and LFCN block to exert a greater analgesic advantage at this reduced level. In contrast, if baseline pain levels are excessively high, the efficacy of combined local nerve blockade may not be as evident. Interestingly, the favourable outcomes in terms of analgesia and recovery may suggest that the process of regaining mobility is less dependent on the combined application of neuroblocking techniques, resulting in negligible differences in the associated subgroup outcomes. Finally, with regard to the surgical approach, whether it is minimally invasive or not, the combined PENG and LFCN block demonstrates robust effectiveness. These findings may indicate that the combination is applicable for various types of hip surgeries, thereby validating the efficacy of the PENG block in conjunction with the LFCN block.

## Limitations

Although the quality of evidence in this study is relatively high, it is notable that only seven RCTs employed a double-blind design, which may introduce a potential risk of bias. In the context of neuroblocking procedures, while operators can maintain full independence and refrain from participation in the research and data analysis, the blinding process for patients demands stringent adherence to protocols. Although some studies reported their blinding mechanisms, the consistency and efficacy of various blinding protocols under a double-blind design remain to be validated further. Furthermore, certain studies that employed nonblinded or nondouble-blind designs may significantly impact the results of this analysis. Additionally, the analyses of multiple indicators in this study exhibited considerable heterogeneity, and the subgroup analyses conducted across different groups did not effectively identify the sources of this heterogeneity. Although this study included twelve RCTs, the limited number of studies and sample sizes for specific indicators constrained further subdivision in the subgroup analyses. Additionally, certain confounding factors, such as whether the block was preoperative or postoperative, whether the surgery was elective, and the approach to arthroplasty, lacked clear subgroup information, which precluded subgroup analyses and consequently impacted the robustness of the findings. Finally, the inclusion of relevant indicators, such as the dose of rescue analgesics employed and the mobility of adductors, was insufficiently addressed in the RCTs, which may have restricted our comprehensive analysis. These factors may contribute to potential bias, particularly concerning results related to pain scores and the use of rescue analgesics. Therefore, additional high-quality studies are warranted to address these concerns.

## Conclusion

In summary, the results of our study suggest that the combined PENG and LFCN block can significantly reduce pain scores and the need for rescue analgesics, prolong the time to first rescue analgesia, decrease the incidence of quadriceps weakness, shorten the time to first mobilization, and substantially lower the occurrence of PONV in patients following hip surgery. Therefore, we recommend the PENG block combined with the LFCN block for pain management in patients after hip surgery. However, additional high-quality, large-scale RCTs are needed to validate our findings.

## Data Availability

The original contributions presented in the study are included in the article/Supplementary Material, further inquiries can be directed to the corresponding author.
