# Supplementary material for: Pericapsular nerve group block combined with lateral femoral cutaneous nerve block for hip surgery: a meta-analysis
Source: Front Pain Res (Lausanne). 2026 Jan 12;6:1723417. doi: 10.3389/fpain.2025.1723417 (PMC12833027; doi:10.3389/fpain.2025.1723417)
Supplement: Supplementary file 1 [file Datasheet1.pdf]

Supplementary Table and Figure

Table S1. Example of search order in Pubmed.

Order	MeSH
1#	lateral femoral cutaneous nerve
2#	pericapsular nerve
3#	1# AND 2#
4#	hip
5#	arthroplasty
6#	fracture
7#	replacement
8#	arthroscopic
9#	replacement
10#	5# OR 6# OR 7# OR 8# OR 9#
11#	4# AND 10#
12#	VAS
13#	visual analog scale
14#	NRS
15#	numerical rating scale
16#	pain score
17#	12# OR 13#
18#	14# OR 15#
19#	16# OR 17# OR 18#
20#	rescue analgesics
21#	quantity
22#	consumption
23#	21# OR 22#
24#	19# AND 23#
25#	quadriceps weakness
26#	first walk
27#	out of bed
28#	26# OR 27#
29#	postoperative nausea and vomiting
30#	PONV
31#	29# OR 30#
32#	19# OR 24# OR 25# OR 28# OR 31#
33#	randomized
34#	controlled clinical trial
35#	randomized controlled trial
36#	retrospective

37#	observational
38#	cross-sectional
39#	33# OR 34# OR 35#
40#	36# OR 37# OR 38#
41#	39# NOT 40#
42#	11# AND 32# AND 41#

Figure S1.

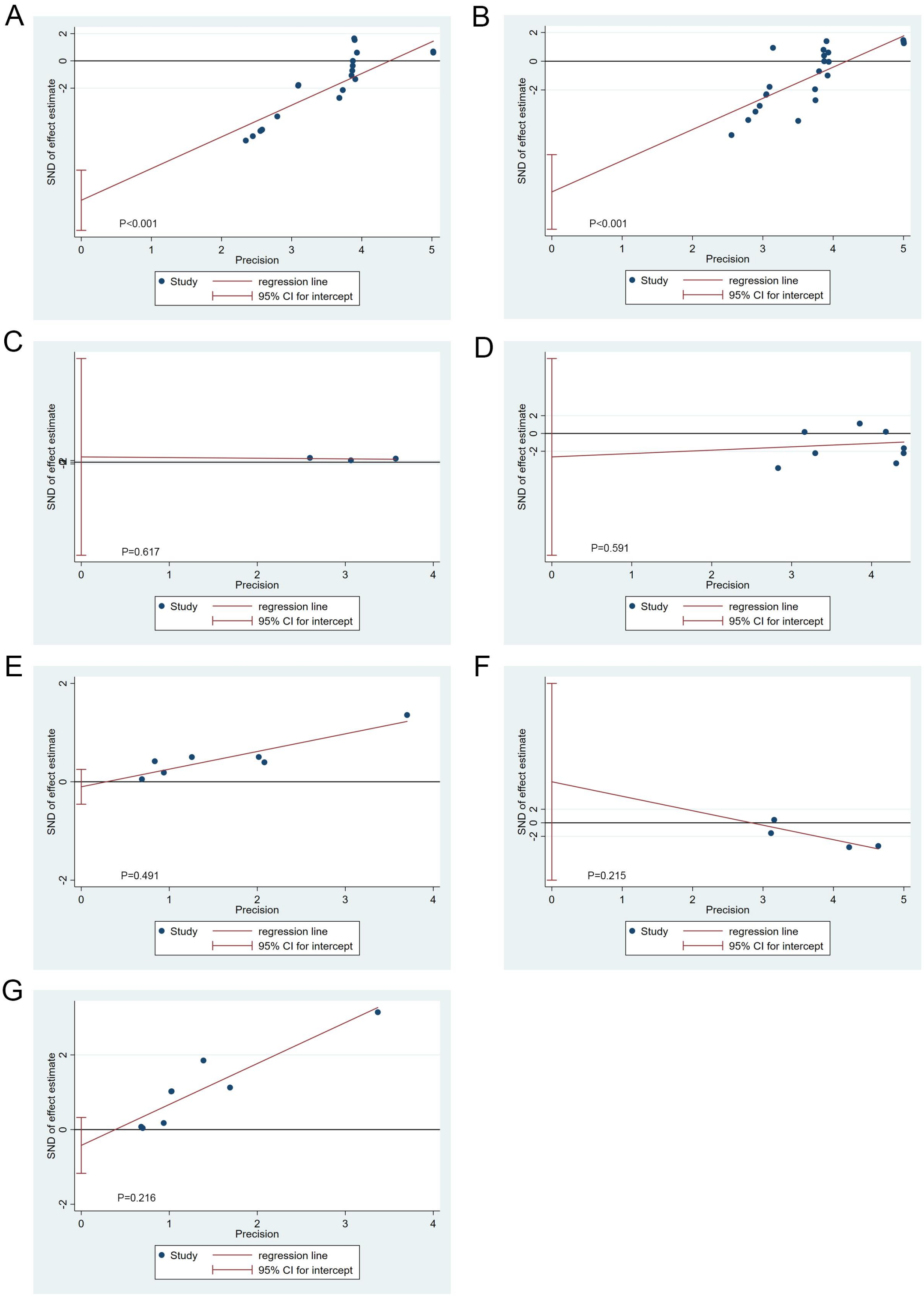

Figure S1. Egger's test of publication bias regarding (A) pain score at rest, (B) pain score at movement, (C) time to first rescue analgesia, (D) consumption of rescue analgesics, (E) the incidence of quadriceps weakness, (F) time to first walk and (G) the incidence of postoperative nausea and vomiting.
